# Trends in Australian knee injury rates: An epidemiological analysis of 228,344 knee injuries over 20 years

**DOI:** 10.1016/j.lanwpc.2022.100409

**Published:** 2022-03-22

**Authors:** Nirav Maniar, Evert Verhagen, Adam Leigh Bryant, David Andrew Opar

**Affiliations:** a School of Behavioural and Health Sciences, Australian Catholic University, Fitzroy, Victoria, Australia; b Sports Performance, Recovery, Injury and New Technologies (SPRINT) Research Centre, Australian Catholic University, Fitzroy, Victoria, Australia; c Amsterdam Collaboration on Health and Safety in Sports, Department of Public and Occupational Health, Amsterdam UMC and Amsterdam Movement Sciences, Amsterdam, the Netherlands; d Centre for Health, Exercise and Sports Medicine, The University of Melbourne, Parkville, Victoria, Australia

## Abstract

**Background:**

Acute knee injuries are a key predisposing risk factor for knee osteoarthritis. Public health interventions require in-depth epidemiological evidence to determine which knee injuries are problematic in critical age and sex demographics.

**Methods:**

Descriptive epidemiological analysis of longitudinal data on knee injuries (July 1998 – June 2018) from the National Hospital Morbidity Database in Australia were studied. The main outcomes where the population-related knee injury frequency, incidence per 100,000 and annual growth rate (%) over the 20-year observation period. Age-group and sex differences were also studied to determine demographic-specific trends.

**Findings:**

228,344 knee injuries were diagnosed over the 20-year analysis period. Significantly rising annual incidences were observed for total knee injuries, anterior cruciate ligament (ACL) injuries and knee contusions in males and females. Posterior cruciate ligament (PCL) injuries and knee dislocations were also rising in females, but not males. Greater annual growth rates were observed for females compared to males for total knee injuries, knee contusions, PCL injuries and knee dislocations. Demographic analysis revealed that the highest annual growth rate in injury incidence (10.4%) was observed for ACL injuries in females aged 5–14 years old.

**Interpretation:**

Increasing annual incidence of knee injuries was observed over the 20-year period. Males have a higher incidence of knee injury per capita than females, but the gap appears to have narrowed over the 20-year analysis period. Younger Australians show a precipitous rise in the annual number of ACL injuries, particularly for females aged 5–14 years. These trends warrant urgent intervention.

**Funding:**

None.

**Research in context**

**Evidence before this study**

Osteoarthritis is one of the most burdensome conditions globally, with most of this burden stemming from knee osteoarthritis. Previous research has shown that a variety of traumatic knee injuries are associated with increased risk of knee osteoarthritis, and thus public health strategies to mitigate their incidence is critical to the management of osteoarthritis related burden. However, such preventative strategies require resources, and thus there is a need for epidemiological studies to determine which knee injuries are most problematic in critical age and sex groups in order to determine priority groups for intervention.

**Added value of this study**

This study has determined that knee injuries have risen in Australia over the last 20-years, and this increase is more precipitous in females compared to males. Our analysis suggests that these increases are largely due to increasing anterior cruciate ligament (ACL) injuries, knee contusions and, in females, posterior cruciate ligament injuries and knee dislocations. People aged 15–29 have the highest incidence of knee injury, but younger individuals aged 5–14 showed the steepest rise in any injury (ACL injuries) over the 20-year analysis period.

**Implications of the available evidence**

This study shows that urgent intervention is required to stop the annual rise in acute knee injuries and to limit the subsequent burden of knee osteoarthritis. Our analysis indicates that public health intervention should target individuals aged 15–29 to obtain the greatest cost-benefit, but attention to young individuals (especially females aged 5–14) is needed to level steep rises in ACL injuries. As rises in injury rates appear to be driven by increased physical activity participation (particularly in young females), it is recommended that national and international public health policy, which currently promote exercise and physical activity in young girls, must also embed knee injury prevention strategies.

Alt-text: Unlabelled box

## Introduction

Osteoarthritis is a highly burdensome condition and a major cause of disability, psychological stress, and poor quality of life.^1^ In Australia, the healthcare costs of osteoarthritis have been estimated to exceed AUD$3.5 billion annually.^2^ Whilst osteoarthritis can affect numerous joints, knee osteoarthritis accounts for approximately 85% of the global burden of the disease.^3^ Whilst knee osteoarthritis can be managed conservatively or surgically,^4^ total knee replacements are increasing rapidly in Australia.^5^ Thus, primary prevention of knee osteoarthritis is critical to manage the increasing burden on the healthcare system.^1^

Whilst the causes of knee osteoarthritis are multifactorial, traumatic knee injury has been identified as a key predisposing factor.^6^ Knees with a previous anterior cruciate ligament (ACL) injury or meniscus injury have 4.2 and 6.3 greater odds, respectively, for developing tibiofemoral osteoarthritis compared to uninjured knees.^6^ Increased risk of tibiofemoral osteoarthritis has also been observed following other knee injuries, such as knee dislocations, contusions, collateral ligament injuries and cartilage tears.^7^ Links with patellofemoral osteoarthritis have also been established for both ACL injuries^8^ and patella dislocations.^9^ As such, limiting the total incidence of traumatic knee injuries should be a public health priority.^10^

Prior research has shown that injury prevention programs are capable of reducing the risk of knee injuries in sporting cohorts.^11^ Achieving a reduction in knee injuries across the population, however, would require substantial public health intervention. Whilst universal implementation of injury prevention programs is appealing, such a strategy would be more costly than targeted interventions. Screening tests may permit the identification of “high risk” individuals and thus permit more targeted and cost-effective interventions, but the poor predictive performance of current screening tests renders them uneconomical.^12^ An alternative approach is to identify the demographic features within the population who sustain specific types of knee injuries. Such an analysis could identify specific knee injuries that occur at higher rates for certain sex and age demographics which, in turn, could inform priority groups for targeted interventions. Additionally, such an analysis may identify which injuries are increasing in incidence over time, thus informing priority areas for public health interventions.

Using publicly available data, this study aimed to (1) determine incidence of knee injuries according to age and sex, (2) report the change in annual frequency and incidence of knee injuries in Australia over the last 20 years, and (3) to use the 20-year trends to estimate the projected burden of knee injuries until the year 2030.

## Methods

### Study design

Longitudinal data for knee injuries were extracted from the National Hospital Morbidity Database (NHMD) of the Australian Institute of Health and Welfare (AIHW). The NHMD comprises episode-level records from admitted patient morbidity data collection systems from public and private hospitals in Australia. The database includes information on the principal diagnosis according to the International Classification of Diseases and Related Health Problems, 10th revision, Australian modification [ICD-10-AM]. For each specific knee injury, the total number of diagnoses, stratified for sex and age group (in 5-year increments), were extracted from publicly available data cubes. Since the data used in this study is publicly available de-identified data, the Australian Catholic University Human Research Ethics Committee granted an exemption from full review (ethics approval number: 2020–225N).

The total number of principal diagnoses for each knee injury subclassification were obtained during a 20-year period from 1 July 1998 to 30 June 2018. This period covers the earliest available diagnosis data classified according to the ICD-10-AM system, to the latest data available at the time of the analysis. Knee injuries were identified by ICD-10-AM 3-digit diagnosis code “S83”, with the exception of knee contusions, which fell under “S80”. Specific knee injuries under the “S83” code were identified by 4-digit and 5-digit diagnosis codes (see Supplementary Table 1 for full details). The specific knee injuries identifiable via 4-digit and 5-digit codes included injuries (ruptures, sprains and strains) to specific knee ligaments (ACL, posterior cruciate ligament (PCL), medial collateral ligament (MCL), and lateral collateral ligament (LCL)), joint dislocations (knee or patella), meniscus tears, articular cartilage tears and other unspecified injuries (unspecified knee sprains, unspecified cruciate or collateral sprains, and injuries to multiple knee structures). For our analysis of total knee injuries, all knee injury diagnoses were included. With respect to our analysis of knee injuries subtypes, unspecified injuries and injuries to multiple structures were not analysed as their definitions were insufficiently specific.

### Statistical analysis

All statistical analysis and data visualisation were conducted using selected packages (“MASS”^13^, “dplyr”^14^, “tidyr”^15^ and “ggplot2”^16^) packages in R (R Core Team, 2020). The overall distribution of knee injury frequency (summed over the 20-year analysis period) and its relationship with population demographics (age and sex) were evaluated visually, using population pyramids.

The annual growth or decline rate in knee injury incidence over the 20-year analysis period (for total knee injuries and each specific knee injury) was evaluated using negative binomial regression, due to overdispersion of the data. Since the annual injury frequency may have increased over the 20-year analysis period due to population growth, we incorporated data from the Australian Bureau of Statistics (ABS) population figures. From a results perspective, this involved expressing annual knee injury incidence per 100,000 population (annual knee injury frequencies are available in Supplementary Fig. S1). To account for population changes in the regression analysis, we included an offset term for the log-transformed population for each given year. We also included an interaction term to determine if growth rates were moderated by sex. These regression models were extrapolated to estimate annual injury incidence until the year 2030. To produce the most accurate estimations, we input predicted populations from ABS (details covered in Supplementary Fig. S2). To evaluate each model's performance, we used 10-fold cross validation to compute the root-mean-square error (Supplementary Table S2).

Similar to previous work,^17^^,^^18^ we further investigated the influence of demographics by calculating sex-specific growth rates for the following age groups: 5–14, 15–24, 25–34, 35–44, and 45+ years. Note that in this analysis, data was occasionally not over dispersed (e.g., articular cartilage tears for 35–44-year-old males); hence, negative binomial regression was not appropriate. In these instances, Poisson regression was used instead. Since negative binomial and Poisson regression coefficients represent the expected change in the log counts for each 1-year increment, the coefficients were back-transformed (i.e., exponentiated) to improve interpretability. Ultimately, the back-transformed coefficients were expressed as the expected percent (%) change and the 95% confidence interval (CI) in the incidence of injury for each annual increment. Statistical significance was set at *p* < 0.05 for all analysis.

### Role of the funding source

Authors have no funding to declare. All authors had full access to the full data in the study and accept responsibility to submit for publication.

## Results

### Total knee injuries

**Figure 1:**
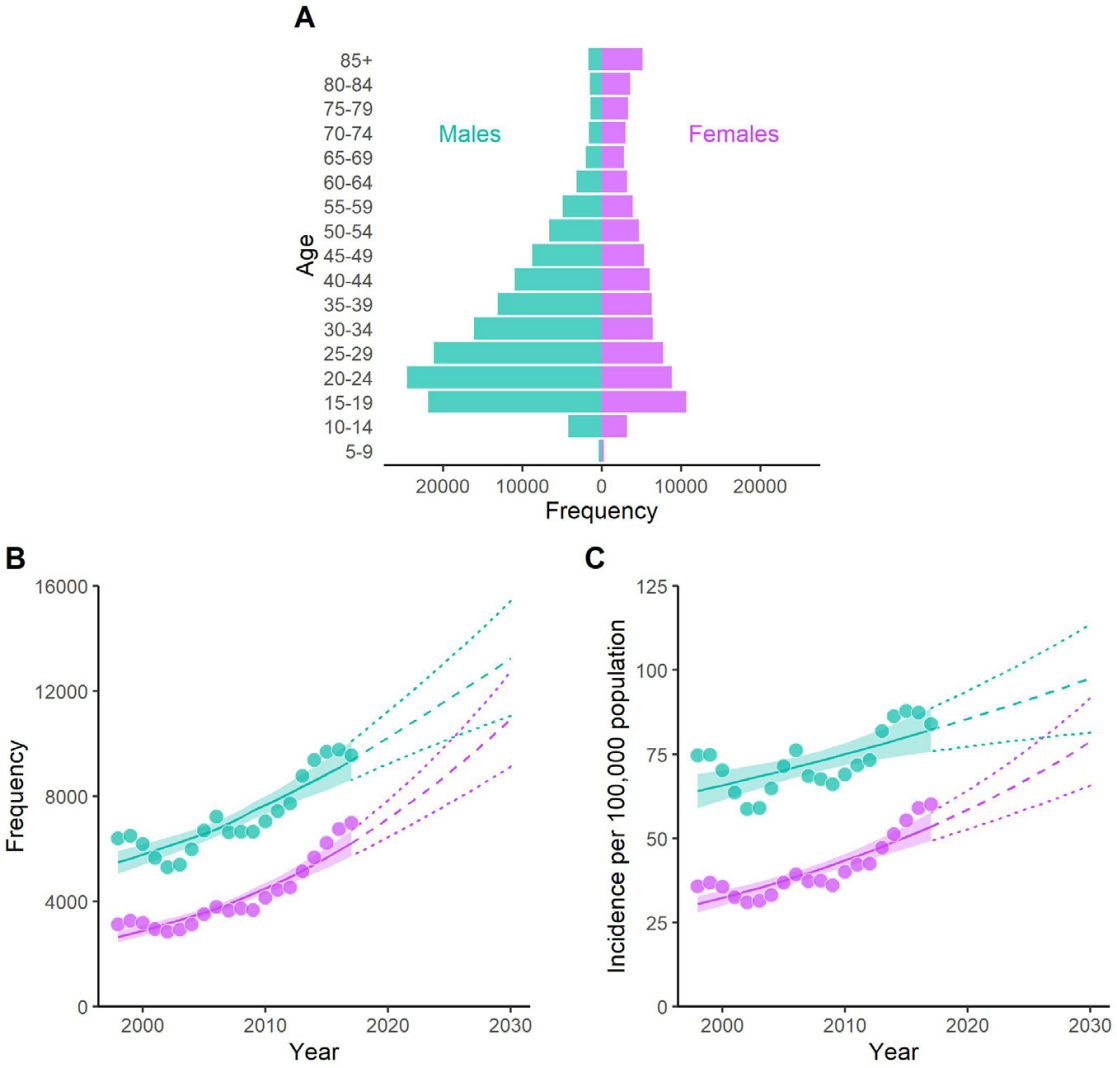
Knee injuries diagnosed in Australian hospitals from 1998-1999 to 2017-2018. Panel A) population pyramid of the total number of acute knee injuries in the 20-year analysis period diagnosed in males (green) and females (purple) aged 5 years and older; panel B) annual knee injury frequency for males and females from the analysis period and extrapolated until the year 2030-2031, panel C) annual knee injury incidence per 100,000 population for males and females for the analysis period and extrapolated until the year 2030-2031. For panels B and C: circles, annual knee injury number or incidence; solid line and shaded region, negative binomial regression model and 95% confidence interval, dashed and dotted lines, extrapolated negative binomial regression model and 95% confidence interval. Note the x-axis value in panels B and C indicate the index year i.e., 2000 represents from July 2000 to June 2001.

Over the 20-year analysis period, there were 228,344 reported knee injury diagnoses, with the majority (63%) of these injuries occurring in males. For both sexes, knee injuries increased in frequency throughout childhood and adolescence, peaking between 15 and 24 years of age, then declining thereafter (Figure 1A). Over the 20-year analysis period, annual knee injury incidence showed significant growth for both males and females (Figure 1B and 1C), with the most recent (2017–2018) annual incidence per capita found to be 83.9 and 60.1 per 100,000 population for males and females, respectively. A significant interaction for sex was observed (*p* = 0.001), suggesting more rapid growth in annual knee injury incidence for females (3.0% per year, 95%CI = 2.3–3.8%) compared to males (1.3% per year, 95%CI = 0.7–2.0%). Based on the annual growth rate from the 20-year analysis period and projected population growth, annual knee injuries for males and females were estimated to reach 13,247 (95%CI = 11,068–15,426) and 10,951 (95%CI = 9140–12,763) by the year 2030–2031 (Figure 1B), respectively. When expressed per capita, the annual knee injury incidence was estimated to reach 97.6 (95%CI = 81.5–113.6) and 78.8 (95%CI = 65.8–91.8) injuries per 100,000 population, for males and females respectively (Figure 1C) by 2030–2031.

### Specific knee injuries

**Figure 2:**
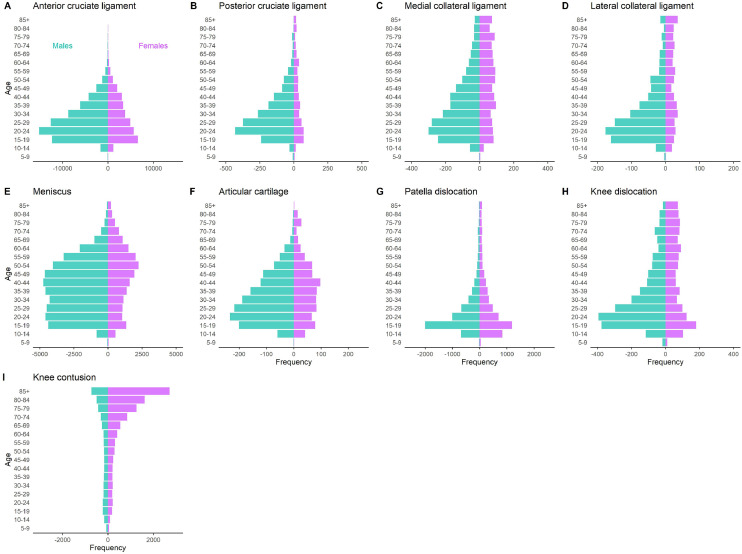
Population pyramid of each specific knee injury diagnosed in Australian hospitals from 1998-1999 to 2017-2018. Each panel shows the total number of injuries diagnosed in the 20-year analysis period in males (green) and females (purple) aged 5 years and older.

**Figure 3:**
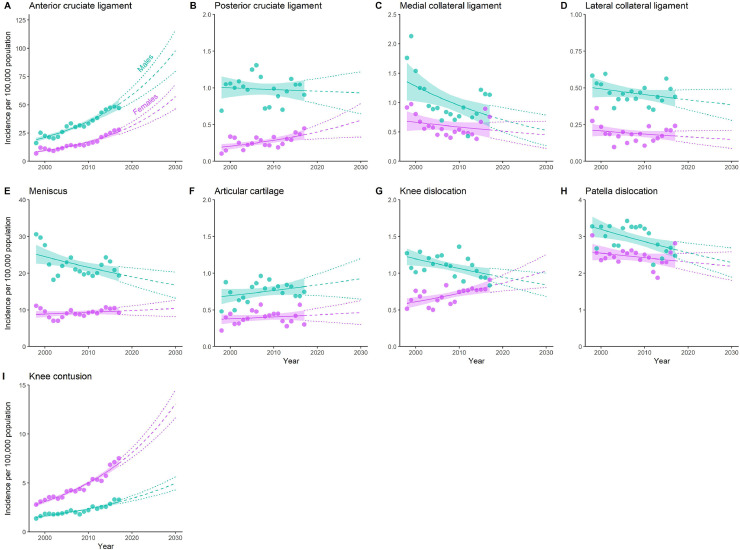
Annual injury incidence (per 100,000 population) for specific knee injuries diagnosed in Australian hospitals in individuals aged 5 years and older from 1998-1999 to 2017-2018. Circles, annual knee injury incidence; solid line and shaded region, negative binomial regression model and 95% confidence interval, dashed and dotted lines, extrapolated negative binomial regression model and 95% confidence interval. Note the x-axis value indicate the index year i.e., 2000 represents from July 2000 to June 2001.

Across all specific knee injuries, males typically had a greater frequency of injury compared to females over the 20-year analysis period (Figure 2), although exceptions did occur in some instances (e.g., knee contusions in individuals 45 years and older). Over the 20-year analysis period, significant annual growth was observed for the incidence of ACL injuries and knee contusions for both males and females (Figure 3). For ACL injuries, annual growth rates were not significantly different between males (5.2% per year, 95%CI = 4.5–6.0%) and females (6.2% per year, 95%CI = 5.3–7.1%), whilst knee contusions displayed a significant interaction (*p* = 0.008) suggesting higher annual growth rates in females (4.9% per year, 95%CI = 4.4–5.4%) compared to males (3.8% per year, 95%CI = 3.2–4.4%). Significant sex interactions were also observed for PCL injuries (*p* = 0.002), knee dislocations (*p* < 0.001) and meniscus tears (*p* = 0.007). In the case of PCL injuries and knee dislocations, this was due to significant annual growth for females (PCL: 3.4%, 95%CI = 1.5–5.4%; knee dislocations: 1.8%, 95%CI = 0.9–2.7%) compared to relatively stable (PCL: -0.2%, 95%CI = -1.5–1.1%) or declining (knee dislocations: -1.2%, 95%CI = -2.0% to -0.3%) annual incidence for males. For meniscus tears, sex interactions were explained by declining annual injury incidence for males (-1.3%, 95%CI = -2.2% to -0.4%) but not females (0.5%, 95%CI = -0.4–1.4%).

### Demographic analysis of knee injuries

**Figure 4:**
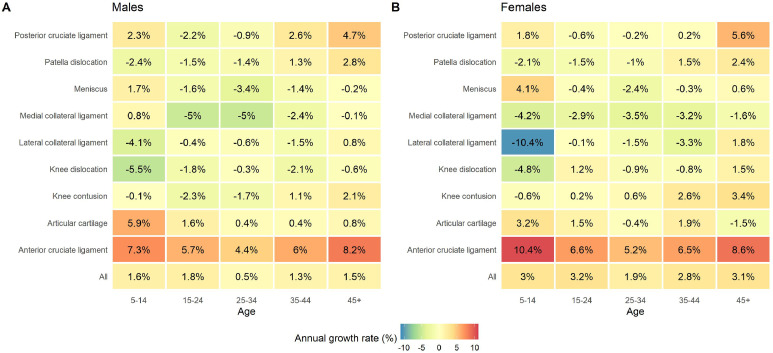
Demographic (age by sex) heat map analysis of the annual growth rate (%) of specific knee injuries diagnosed in individuals aged 5 years and older in Australian hospitals from 1998-1999 to 2017-2018. Panel A) males; panel B) females. Growth rates were determined as the exponentiated co-efficient from negative binomial or Poisson regression (depending on the distribution of the data).

Across all age groups, growth rates for knee injury incidence per capita were typically greater in females compared to males (Figure 4). Total knee injury annual growth rates were highest in the 15–24-year-old age groups for females (3.2% per year, 95%CI = 2.1–4.3%) and males (1.8% per year, 95%CI = 1.1–2.5%) compared to other age groups. The greatest annual growth rate observed for any injury or demographic was for ACL injuries in 5–14-year-old females (10.4% per year, 95%CI = 9.3–11.6%). For females, this age group also had the greatest annual growth rate for injuries to the meniscus (4.1% per year, 95%CI = 2.4–5.8%), but also had the greatest annual declining rates for knee dislocations (-4.8% per year, 95%CI = -8.8 to -0.6%), and LCL injuries (-10.4% per year, 95%CI = -18.1 to -2.9%) compared to other age groups. Older individuals (aged 45 years and older) demonstrated higher annual growth rates for PCL injuries (females, 5.6% per year, 95%CI = 0.9–10.6%; males, 4.7% per year, 95%CI = 1.9–7.8%) and knee contusions (females, 3.4% per year, 95%CI = 1.7–5.3%; males, 2.1% per year, 95%CI = 0.2–4.0%) compared to other age groups.

## Discussion

This study is the first to investigate 20-year knee injury diagnosis trends in Australian hospitals. Our key findings show that (1) total annual knee injuries have increased substantially over the last 20-years, owing to a growth in ACL injuries, knee contusions, and (in females) PCL injuries and knee dislocations; (2) males have greater overall incidence of knee injury per capita than females, but this gap has narrowed over the 20-year analysis period owing to larger growth rates in the annual knee injury incidence in females; and (3) ACL injuries have the fastest annual growth rate of all knee injuries, especially in young (5–14-year-old) females. Without intervention, these trends suggest that the burden of knee injuries is projected to increase. Given the well-established link between knee injuries and subsequent knee osteoarthritis, our findings have substantial implications for public health prioritizations.

Despite the novelty of our approach, indirect support for some of our key findings is available from other literature. For example, our analysis found a relatively high annual growth rate for ACL injury incidence in Australia of 10.4% and 7.3% per year, for 5–14 years-old females and males, respectively. A previous investigation of ACL-reconstruction rates in Australia from 2000 to 2015 showed similar annual growth rates of 8.8% and 7.7% per year, for 5–14 years-old females and males, respectively.^17^ An analysis of paediatric ACL injuries in Finland also showed that young females (aged 13–15 years) showed the largest increase in ACL injury incidence over an 18-year study period, compared to other age/sex groups.^19^ Additionally, data from Southern Sweden^20^ and the United States^21^ showed similar trends in knee injury incidence across the lifespan, whereby knee injury incidence typically increased during childhood, peaked between the ages of 15–24 years, and gradually declined thereafter. Our work provides a substantial extension of this previous work, demonstrating that the incidence of a variety of knee injuries, many of which have been associated with subsequent knee osteoarthritis,^7^ and are increasing over time in a demographic-specific manner.

### Consequences of current trends

Of the knee injuries examined in this analysis, the annual incidence of ACL injuries appears to be increasing most rapidly. Based on our estimations, the annual number of ACL injuries is expected to more than double by 2030–2031 compared to 2017–2018 levels, reaching an incidence of 77.2 per 100,000 population, if no action is taken to reduce the incidence of injury. Although this incidence would be more than 1.6-fold higher than the ACL injury incidence in any other country reported in a 2012 systematic review,^22^ it is unknown if the annual ACL injury incidence in other countries are also expected to increase by the year 2030. Since ACL injury rates may be rising in other countries, further research is warranted to determine the extent of the problem globally. Increasing rates of ACL injury are particularly alarming, as the injury is associated with substantial financial burden owing to its common treatment via surgical intervention and relatively long rehabilitations periods (∼1 year).^23^^,^^24^ Based on an estimated direct cost of A$14,830 (US$11,154) for the surgery and rehabilitation associated with each ACL injury,^24^ the estimated cost of ACL injuries in the year 2030–2031 would amount to an estimated A$314,946,148 (US$236,874,100). Although not all ACL injuries are treated via surgical intervention, this estimate is likely still very conservative, given that the data from the current study (derived from hospitals) would underestimate the true population incidence of ACL injuries since not all ACL injuries are diagnosed in hospitals. Additionally, this estimate does not account for indirect costs (e.g., from persistent knee disability), which can be substantial.^23^

Perhaps the most insidious of consequences, knees with a history of injury have a greater risk of developing knee osteoarthritis. Whilst this link is well established for injuries to the ACL and meniscus,^25^ recent work has shown similar relationships for other injuries to the knee, including articular cartilage tears, collateral ligament injuries, knee contusions and knee dislocations.^7^ The present analysis revealed that the annual incidence of all these injuries (except for collateral ligament injuries) were observed to be growing over time in several demographics, most notably for young females (Figure 4). Thus, the increasing incidence of these injuries is likely to yield substantial burden to the public health system, and therefore warrants urgent intervention.

### Knee injuries across the lifespan

Our data revealed a distinct trend of knee injuries across the lifespan, whereby knee injury frequency increases over childhood, peaks between 15 and 24 years, and gradually declines thereafter. Whilst this suggests that younger individuals are at greater risk of knee injury, it is important to recognise that our analysis, nor prior population analysis conducted in Southern Sweden^20^ or the United States,^21^ accounted for exposure to sporting or other “high risk” tasks. Therefore, differences in the incidence of knee injuries across the lifespan may be due to different exposures to “high risk” activities. Results from Australia's annual physical activity survey^26^ support this, as participation rates in “high risk” sports (i.e., sports requiring frequent changes of direction and landing tasks) over the lifespan tend to resemble the trends that we observed in knee injuries (Figure 2). For example, swimming (a “low risk” activity) is the most popular activity type throughout early childhood, but participation rates in “high risk” sports tend to increase as children get older. At age 14, participation rates in swimming are exceeded by soccer, basketball, netball and Australian Rules football. Into adulthood, participation rates in these “high risk” sports gradually decline, whilst recreational gym use and walking increase in popularity with increasing age.^26^

### Sex differences

Differences in exposure to “high risk” activities likely also explain our observation of greater knee injury incidence in males compared to females. Prior studies have shown that, when sports exposure is accounted for, females have a higher incidence rate of knee injuries compared to males. With respect to ACL injuries, this risk is 3.4-fold greater in females compared to males.^27^ However, males in Australia show greater participation rates in “high risk” sports, such as soccer, basketball and Australian Rules football,^26^ which likely explains the greater knee injury incidence in males in our analysis. Population analysis from the United States also supports this, showing that sports and recreational activities accounts for 62% of knee injuries presenting to emergency departments in males, whilst the same cause is cited in just 36% of cases in females.^18^

Perhaps the most noteworthy sex difference was a greater annual growth rate of knee injury incidence in females compared to males (Figure 4). To our knowledge, our analysis is the first to report such a trend in a variety of knee injuries, thus revealing an important public health concern. One possible explanation for this trend could be increasing female participation in “high risk” sporting activities over the analysis period. Whilst data was not available to directly confirm this in our analysis, data from the Australian state of Victoria provides some support for this hypothesis, showing that female sports participation has increased from 2011 to 2016.^28^ Specifically, data showed that whilst sports participation rates are increasing for both young females and males, young females showed a greater proportional increase compared to other groups (i.e., young males and older individuals).^28^ In fact, females aged 5–9 years had doubled participations rates in 2016 compared to 2011, and these trends may explain the notably high annual growth rates observed for some knee injuries that commonly occur in sporting tasks (e.g., ACL injuries) in this demographic group. Whilst the reasons for these increasing participation rates in females is multi-factorial, prior work suggests that more pathways for females (at the elite and grass roots level) combined with greater media coverage of women's sport may underlie the community level increases in female sport participation.^28^ Indeed, numerous initiatives exist within Australia to either solely or in part to encourage greater female participation in sports, such as the National Sports Plan^29^ the “Girls Make Your Move” campaign^30^ and the Female Performance and Health Initiative.^31^ At a global level, the World Health Organisation (WHO) Global Plan on Physical Activity,^32^ United Nations Women Sports for Generation Equality Framework^33^ and the International Olympic Committee (IOC) Women in Sport Commission^34^ all promote increasing opportunity for girls and women to increase physical activity and/or sports participation levels. These initiatives serve an important societal goal of targeting key barriers to physical activity and sports participation in females. However, none of these initiatives make direct reference to knee injury prevention, despite the well-established greater risk of knee injury in females in numerous sports.^27^ Our analysis suggests that greater consideration need to be given to knee injury prevention as part of these initiatives, as females (especially young females) may have unique vulnerabilities to knee injury.

### Solutions

Recent meta-analyses have shown that injury prevention programs can reduce the incidence of ACL injuries by 53%^11^ and knee injuries by 37%.^35^ Additionally, these programs are relatively simple to implement and can be incorporated as part of the general warm up routine prior to sports participation. Although there are associated costs, recent analysis has shown preventative programs are cost effective when universally applied to a sporting cohort.^12^ Of note, this study also found that the use of screening programs that target only those identified as “at risk” were not cost-effective, owing to the relatively poor predictive performance of screening tests. From a practical standpoint, screening tests may also be difficult to implement at a population level. Consequently, universal implementation of knee injury prevention programs is more favourable at a public health level, and our population analysis (rather than screening) can be used to prioritise the allocation of resources, by identifying the specific knee injuries that need targeting in particular demographic groups.

Our analysis suggests that it is imperative that public health interventions aiming to reduce the societal burden of injury target the prevention of ACL and cartilage (both meniscus and articular) injuries in young Australians. Australians aged 15–29 tend to have the highest incidence of knee injury, and thus implementation of injury prevention programs to this demographic is expected to yield the greatest cost-benefit. The steep rise in annual knee injuries in children, especially females aged 5–14, also warrants urgent attention to prevent an impending public health issue, owing to the previously established associations of ACL and knee cartilage injuries with subsequent knee osteoarthritis.^7^ Public health strategy may involve promoting greater awareness of knee injuries and embedding evidence-based injury prevention programs in national^29^, ^30^, ^31^ and international policy,^32^, ^33^, ^34^ which promote physical activity and sports participation in females but currently lack any knee injury prevention strategies. Additionally, promotion of sports participation with concurrent failure to provide appropriate infrastructure support, such as access to facilities, may contribute to the rising incidence of knee injuries in females. Finally, the establishment of accurate knee injury surveillance at a national and international level is critical to identifying and addressing knee injury trends in different countries.

### Limitations

The nature of the data used in this analysis presents several limitations. Firstly, we relied on data of the NHMD. Whilst this essentially covers all public and private hospitals in Australia, not all knee injuries are diagnosed in hospitals. This limitation would likely not influence our analysis of annual growth rates, but it is important not to compare the observed incidence of some injuries to others (e.g., ACL injuries to meniscus tears), as those with less severe knee injury types are less likely to attend a hospital. This limitation also explains the lower observed incidence of ACL injuries in males and females (e.g., 47.2 and 28.0 per 100,000 population, respectively, in 2017–2018), compared to the incidence of ACL reconstruction reported by Zbrojkiewicz^17^ (101.6 and 53.4 per 100,000 population in 2014–2015), as reconstructive surgeries would have better coverage in hospitals. Thus, it is important to recognise that our estimates of knee injury population incidence are conservative, although we have no reason to believe that this limitation would influence our key conclusions pertaining to relative trends (i.e., annual growth rates and comparisons across age and sex). It is also worth noting that the potential influence of missing data and variations in data collection (e.g., changes in hospital admission policy) cannot be discounted. Whilst we accept this limitation, these potential issues have been documented in data quality statements,^36^ and are either inconsequential to our analysis (i.e., not related to knee injuries) or are more likely to have resulted in an underestimation of numbers in more recent years. Collectively, this would suggest that our calculated growth rates are unlikely to have been overestimated, and we therefore believe our conclusions to be robust. Knee injuries could also have been misdiagnosed; however, this is unlikely to have occurred in a pattern systematic enough (e.g., for specific demographics) to influence our analysis. Finally, our predictive estimations of knee injury incidence for subsequent years were computed from trends over the preceding 20-years and limited by data provided by the NHMD (i.e., age and sex) and ABS population predictions. In addition to the inherent limitation of extrapolations using such data, significant events such as the COVID-19 pandemic are likely to influence knee injury incidence and population growth from the year 2020 onward, and we thus acknowledge the limitations of our predictive estimations and encourage more comprehensive data acquisition for future analysis.

## Conclusion

Increasing annual incidence of knee injuries were observed over the 20-year analysis period, largely due to increasing ACL injuries, knee contusions and, in females, PCL injuries and knee dislocations. Males have higher incidence of knee injury per capita than females, but the gap appears to have narrowed over time owing to greater growth rates in the annual knee injury incidence in females. Changes in annual injury incidence varied according to the specific knee injury type, sex and age group, with younger Australians (e.g., females aged 5-14 years) showing a precipitous rise in the annual incidence of ACL injuries. Left unaddressed, the trends suggest the burden of knee injuries is projected to increase, especially for females. These findings have substantial implications for the burden of early-onset knee osteoarthritis in subsequent years, and thus warrant urgent attention at a public health level. It is recommended that national and international policies that aim to promote sports and physical activity participation (especially in females) also embed knee injury prevention strategies. Furthermore, analysis of data in other countries is warranted to determine if similar trends can be observed.

## Declaration of interests

All authors have completed the Unified Competing Interest form (available on request from the corresponding author) and declare: no support from any organisation for the submitted work; no financial relationships with any organisations that might have an interest in the submitted work in the previous three years, no other relationships or activities that could appear to have influenced the submitted work.

## Appendix Supplementary materials

Image, application 1
