# Supplementary material for: Trends in Australian knee injury rates: An epidemiological analysis of 228,344 knee injuries over 20 years
Source: Lancet Reg Health West Pac. 2022 Mar 22;21:100409. doi: 10.1016/j.lanwpc.2022.100409 (PMC8956823; doi:10.1016/j.lanwpc.2022.100409)
Supplement: Supplementary file 1 [file mmc1.docx]

Supplementary Information

This document contains information supplementary to the article: “Trends in Australian knee injury rates: an epidemiological analysis of 228,344 knee injuries over 20 years”. This supplementary information includes:

1. International Classification of Diseases and Related Health Problems, 10th revision, Australian modification [ICD-10-AM] diagnostic codes used to identify the relevant knee injuries (Supplementary Table S1)
2. Results of 10-fold cross validation for all predictive negative binomial regression models
3. Annual knee injury frequencies (Supplementary Figure S1)
4. Australian Bureau of Statistics (ABS) population estimates that were used in the extrapolation analysis (Supplementary Figure S2).

Supplementary Table S1: The 4- and 5-digit International Classification of Diseases and Related Health Problems, 10th revision, Australian modification [ICD-10-AM] diagnostic codes for the knee injury sub-classification.

| 4-digit code | 5-digit code | Injury |
| --- | --- | --- |
| S80.0 | - | Knee contusion |
| S83.0 | - | Dislocation of patella |
| S83.1 | - | Dislocation of knee |
| S83.2 | - | Tear of meniscus |
| S83.3 | - | Tear of articular cartilage |
| S83.4 | S83.41 | Sprain and strain of lateral collateral ligament |
|  | S83.42 | Sprain and strain of medial collateral ligament |
|  | S83.43 | Rupture of lateral collateral ligament |
|  | S83.44 | Rupture of medial collateral ligament |
| S83.5 | S83.51 | Sprain and strain of anterior cruciate ligament |
|  | S83.52 | Sprain and strain of posterior cruciate ligament |
|  | S83.53 | Rupture of anterior cruciate ligament |
|  | S83.54 | Rupture of posterior cruciate ligament |

Supplementary Table S2. Root mean square error (number of injuries) of negative binomial regression models derived from 10-fold cross validation.

| Knee injury | Root mean square error |
| --- | --- |
| All knee injuries | 567 |
| Anterior cruciate ligament | 222 |
| Posterior cruciate ligament | 14 |
| Medial collateral ligament | 28 |
| Lateral collateral ligament | 7 |
| Meniscus | 218 |
| Articular cartilage | 12 |
| Patella dislocation | 12 |
| Knee dislocation | 32 |
| Knee contusion | 29 |

Root mean square error was rounded to the nearest injury. Note that the root mean square error (RMSE) was computed according to the following equation: $RMSE=\sqrt{\frac{1}{n}\sum_{i-1}^{n} \left( y_{i}-\hat{y}_{i} \right)^{2}}$ where $y_{i}$ are the observed values, and $\hat{y}_{i}$ are the fitted values.


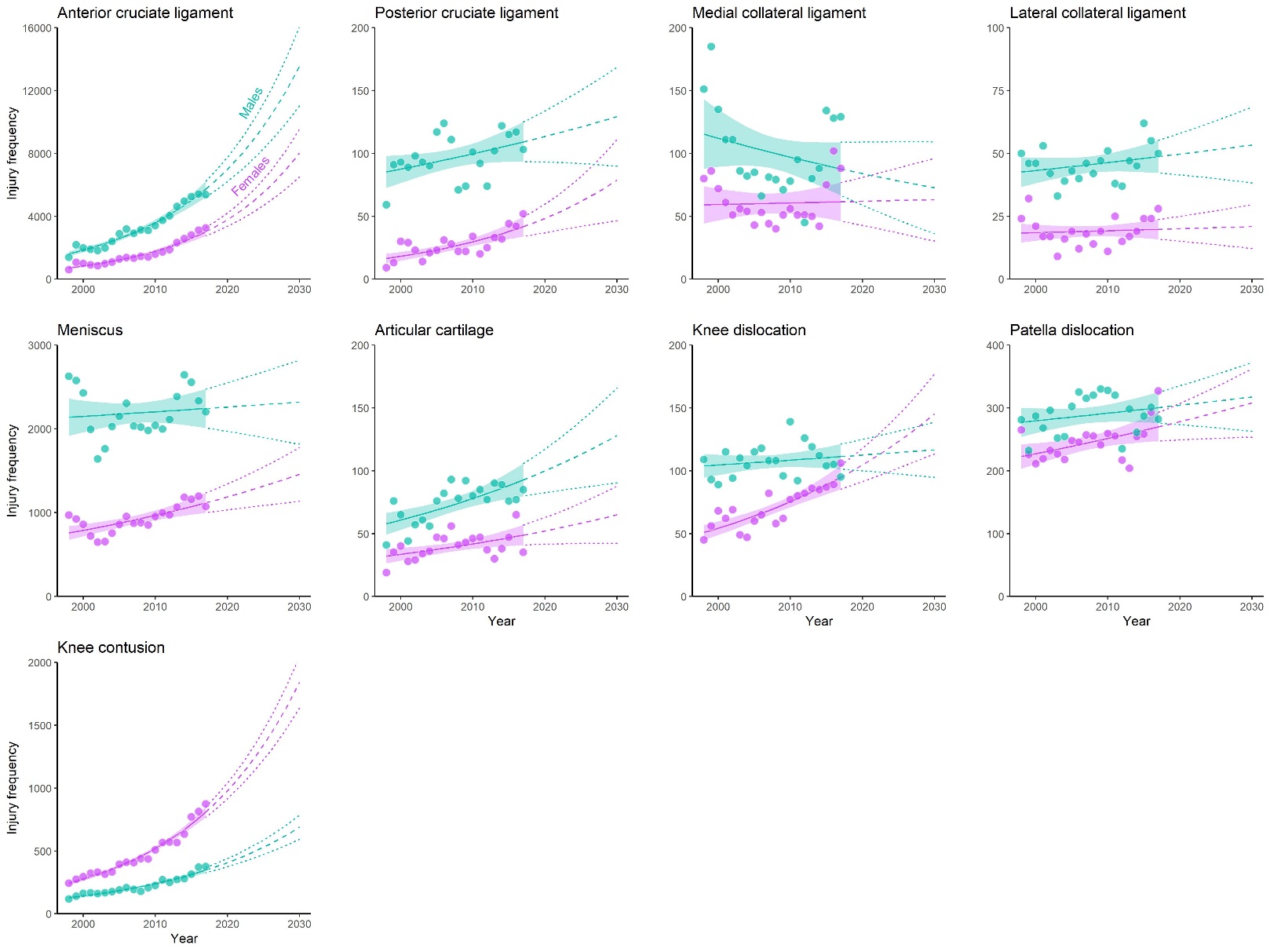


Supplementary Figure S1. Annual injury frequency for specific knee injuries diagnosed in Australian hospitals in individuals aged 5 years and older from 1998-1999 to 2017-2018. Circles, annual knee injury frequency; solid line and shaded region, negative binomial regression model and 95% confidence interval, dashed and dotted lines, extrapolated negative binomial regression model and 95% confidence interval. Note the x-axis value indicate the index year i.e., 2000 represents from July 2000 to June 2001.


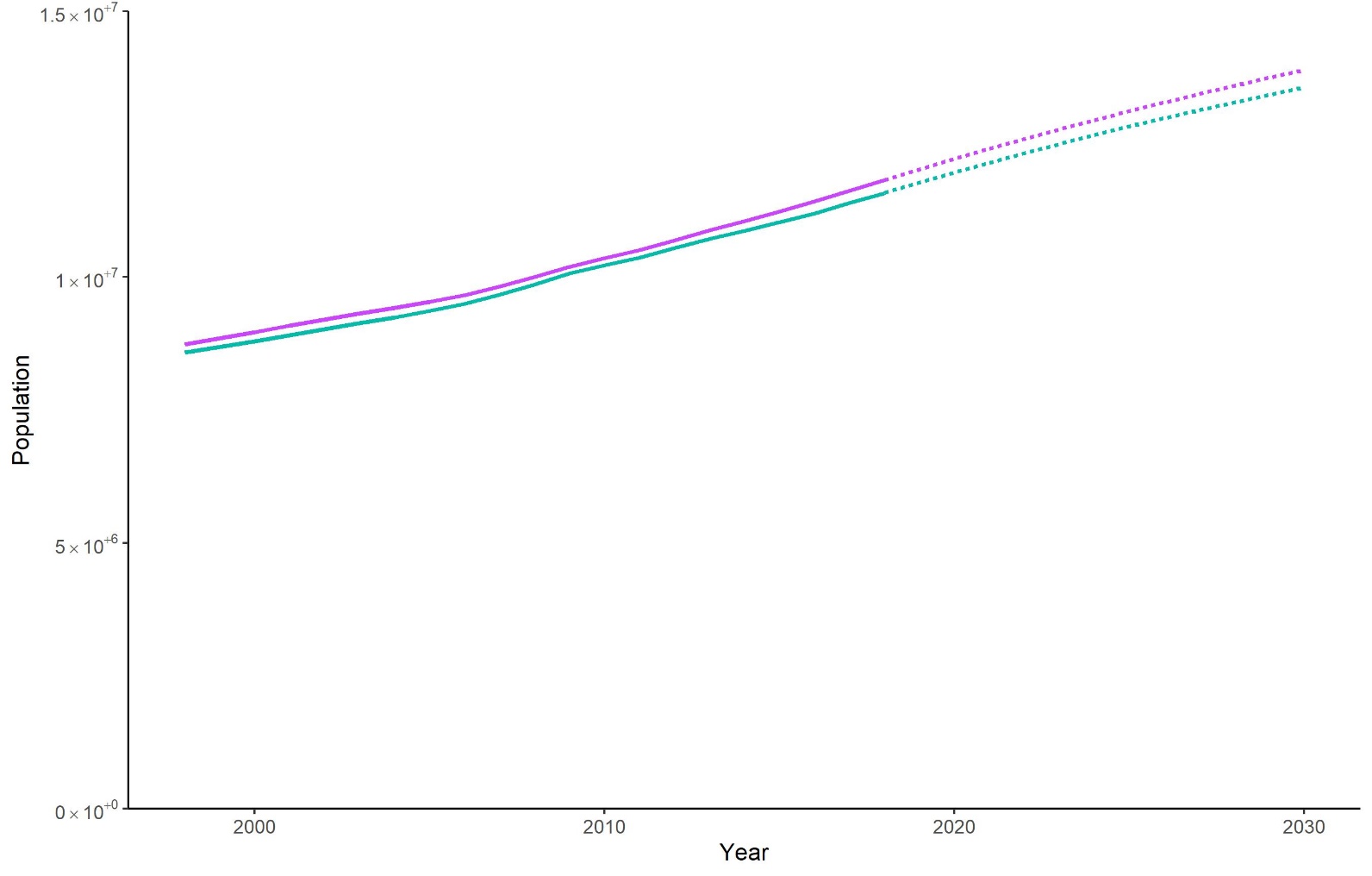


Supplementary Figure S2. Australian population predictions for males (green) and females (purple) aged 5 years and over until the year 2030-2031 based on Australian Bureau of Statistics (ABS) projections. Solid lines, observed population; dotted line, predicted population. Note that population estimates only included persons aged 5 year and older for consistency with knee injury data.
